# Evaluating Different Equating Setups in the Continuous Item Pool Calibration for Computerized Adaptive Testing

**DOI:** 10.3389/fpsyg.2019.01277

**Published:** 2019-06-06

**Authors:** Sebastian Born, Aron Fink, Christian Spoden, Andreas Frey

**Affiliations:** ^1^Department of Research Methods in Education, Institute of Educational Science, Friedrich Schiller University Jena, Jena, Germany; ^2^Educational Psychology: Measurement, Evaluation and Counseling, Institute of Psychology, Goethe University Frankfurt, Frankfurt, Germany; ^3^German Institute for Adult Education, Leibniz Centre for Lifelong Learning, Bonn, Germany; ^4^Faculty of Educational Sciences, Centre for Educational Measurement, University of Oslo, Oslo, Norway

**Keywords:** computerized adaptive test, item response theory, equating, continuous calibration, simulation

## Abstract

The increasing digitalization in the field of psychological and educational testing opens up new opportunities to innovate assessments in many respects (e.g., new item formats, flexible test assembly, efficient data handling). In particular, computerized adaptive testing provides the opportunity to make tests more individualized and more efficient. The newly developed continuous calibration strategy (CCS) from Fink et al. (2018) makes it possible to construct computerized adaptive tests in application areas where separate calibration studies are not feasible. Due to the goal of reporting on a common metric across test cycles, the equating is crucial for the CCS. The quality of the equating depends on the common items selected and the scale transformation method applied. Given the novelty of the CCS, the aim of the study was to evaluate different equating setups in the CCS and to derive practical recommendations. The impact of different equating setups on the precision of item parameter estimates and on the quality of the equating was examined in a Monte Carlo simulation, based on a fully crossed design with the factors common item difficulty distribution (bimodal, normal, uniform), scale transformation method (mean/mean, mean/sigma, Haebara, Stocking-Lord), and sample size per test cycle (50, 100, 300). The quality of the equating was operationalized by three criteria (proportion of feasible equatings, proportion of drifted items, and error of transformation constants). The precision of the item parameter estimates increased with increasing sample size per test cycle, but no substantial difference was found with respect to the common item difficulty distribution and the scale transformation method. With regard to the feasibility of the equatings, no differences were found for the different scale transformation methods. However, when using the moment methods (mean/mean, mean/sigma), quite extreme levels of error for the transformation constants *A* and *B* occurred. Among the characteristic curve method the performance of the Stocking-Lord method was slightly better than for the Haebara method. Thus, while no clear recommendation can be made with regard to the common item difficulty distribution, the characteristic curve methods turned out to be the most favorable scale transformation methods within the CCS.

## Introduction

The shift to using digital technology (e.g., laptops, tablets, and smartphones) for psychological and educational assessments provides the opportunity to implement computer-based state-of-the-art methods from psychometrics and educational measurement in day-to-day testing practice. In particular, computerized adaptive testing (CAT) has the potential to make tests more individualized and to enhance efficiency (e.g., Segall, 2005). CAT is a method of test assembly that uses the responses given to previously presented items for the selection of the next item (e.g., van der Linden, 2016), whereby the item that satisfies a statistical optimality criterion best is selected from a precalibrated item pool. Therefore, the calibrated item pool is an essential and important building block in CAT (e.g., Thompson and Weiss, 2011; He and Reckase, 2014). A set of items is called a calibrated item pool if the item characteristics, such as item difficulty and item discrimination, were estimated on the basis of an item response theory (IRT; e.g., van der Linden, 2016) model beforehand. However, in some contexts, such as higher education, clinical diagnosis, or personnel selection, the item pool calibration for CAT often poses a critical challenge because separate calibration studies are not feasible, and sample sizes are too low to allow for stable item parameter estimation.

To overcome this problem, Fink et al. (2018) proposed a continuous calibration strategy (CCS), which enables a step-by-step build-up of the item pool across several test cycles during the operational CAT phase. In the context of the CCS a test cycle is understood as the whole test procedure including steps like test assembly, test administration and analysis of test results. As the item parameter estimates of existing and new items are continuously updated within the CCS, equating is a critical factor to enable interchangeable score interpretation across test cycles. The equating procedure implemented in the CCS is based on a common-item non-equivalent group design (Kolen and Brennan, 2014) and is carried out in four steps: (1) common item selection, (2) scale transformation, (3) item parameter drift (IPD; e.g., Goldstein, 1983) detection, and (4) fixed common item parameter (FCIP; e.g., Hanson and Béguin, 2002) calibration.

In their study, Fink et al. (2018) evaluated the performance of the CCS for different factors (sample size per test cycle, calibration speed, and IRT model) with respect to the quality of the person parameter estimates. Although the results were promising, two issues remained open. First, the study of Fink et al. (2018) was conducted under ideal conditions (i.e., constant ability distribution of the examinees across test cycles). Second, despite the importance of the equating procedure in the CCS, its performance with respect to different setups of the procedure (i.e., selection of common items, scale transformation method, item drift detection) was not investigated in detail. For example, it became apparent that the CCS did not work as intended for very easy or very difficult items when using small sample sizes (i.e., 50 or 100 examinees) per test cycle. In these cases, item parameter estimates were biased due to a few inconsistent responses, with the consequence that these items were no longer selected by the adaptive algorithm in the following test cycles. Therefore, it was not possible to continuously update the item parameter estimates for these items.

Against this background, the aim of the present study was to investigate the performance of the equating procedure for different setups conducted under more realistic conditions (i.e., examinees’ average abilities and variance differ between test cycles). The remainder of the article is organized as follows: First, we provide the theoretical background for the present study by introducing the underlying IRT model and by describing the CCS. Next, we discuss both the previously implemented equating procedure and alternative specifications. Then, we examine the performance of different setups of the different equating procedures in a simulation. Finally, we discuss the results and make recommendations for the implementation of the CCS.

## Theoretical Background

### IRT Model

The IRT model used in this study was the two-parameter logistic (2PL) model (Birnbaum, 1968) for dichotomous items. The 2PL model defines the probability of a correct response u_ij_ = 1 of examinee j = 1…N with a latent ability level 𝜃_j_ to an item i by the following model, whereby *a*_i_ is the discrimination parameter and *d*_i_ is the easiness parameter of item *i*:

(1) $$P\left(u_{\text{ij}}=1|\theta_{\text{j, }a_{i},d_{i}}\right)=\frac{\exp(a_{i}\theta_{\text{j}}+d_{i})}{1+\exp(a_{i}\theta_{\text{j}}+d_{i})},$$

In the traditional IRT metric where *a*_i_𝜃_j_ + *d*_i_ = *a*_i_ (𝜃_j_ – *b*_i_), the *a*_i_ parameters will be the identical for these parametrizations, while the item difficulty parameter *b*_i_ is calculated as *b*_i_ = -*d*_i_/*a*_i_.

### Continuous Calibration Strategy

In the following paragraphs, we briefly outline the CCS as introduced by Fink et al. (2018) and detail the equating procedure implemented. The CCS consists of two phases, a non-adaptive *initial phase* and a partly adaptive *continuous phase*. In the initial phase, which is the first test cycle of the CCS, the same items are presented to all examinees and only the item order can vary between examinees. In the continuous phase, the tests assembled consist of three types of item clusters (calibration cluster, linking cluster, adaptive cluster), whereby a cluster is comprised of several items. Each type of cluster has a specific goal. The calibration cluster offers the opportunity to include new items in the existing item pool, the linking cluster utilizes common items to allow a scale to be established across test cycles, and the adaptive cluster aims at the enhancement of measurement precision. The items in the calibration and the linking clusters are the same for all examinees and are administered sequentially, whereas the items in the adaptive cluster can differ between examinees due to the adaptive selection algorithm. Each test cycle in the continuous phase can be broken down into seven steps: (1) common item selection for the linking cluster, (2) test assembly and test administration, (3) temporary item parameter estimation, (4) scale transformation of the common items, (5) IPD detection for the common items, (6) FCIP calibration, and (7) person parameter estimation. The equating procedure consists of four of these steps, which will be detailed in the following four paragraphs. The first three steps of the equating procedure serve as quality assurance of the common items to ensure feasible equating in the fourth step.

In the *common item selection*, items that have already been calibrated in the previous test cycles are selected as common items for the linking cluster. To ensure that the common items represent the statistical characteristics of the item pool (Kolen and Brennan, 2014), such as the range of the item difficulty, the items are assigned to five categories (very low, low, medium, high, and very high) based on their easiness parameters *d*_i_. Fink et al. (2018) selected the items from the categories in such a way that the difficulty distribution of the common items corresponded approximately to a normal distribution. Beside the representation of the statistical item pool characteristics it is important that the common items adequately reflect the content of the item pool. This can be done by using content balancing approaches (e.g., van der Linden and Reese, 1998; Cheng and Chang, 2009; Born and Frey, 2017) within the common item selection and within the adaptive cluster.

After test assembly and test administration, the parameters for the common items are estimated based on the responses of the current test cycle. In the second step of the equating procedure, a *scale transformation* of the common items has to be conducted, because the ability distribution of the examinees usually differs between test cycles and, therefore, the item parameter estimates obtained are not directly comparable across cycles. The comparability of the parameter estimates is a necessary condition to check whether the common items are affected by IPD. For this reason, scale transformation methods (e.g., Marco, 1977; Haebara, 1980; Loyd and Hoover, 1980; Stocking and Lord, 1983) are important for the equating procedure. Fink et al. (2018) used the mean/mean method (Loyd and Hoover, 1980) for the scale transformation.

As IPD of item parameters may have a serious impact on equating results such as scaled scores and passing rates (Hu et al., 2008; Miller and Fitzpatrick, 2009), the *IPD detection* as the third step of the equating procedure is important if the method is to operate optimally. A number of tests for IPD can be used in IRT-based equating procedures, such as the Lord’s χ^2^-test (Lord, 1980) and the likelihood-ratio test (Thissen et al., 1988). In an iterative process of scale transformation and testing for IPD, common items that show significant IPD are excluded from the final set of common items. The iterative purification continues as long as at least one of the remaining common items shows significant IPD or less than two common items are left. The rationale behind the latter stopping rule is that at least two link items are necessary to keep the scale comparable across test cycles. Nevertheless, it should be mentioned that with a smaller number of link items, the equating procedure is more prone to sampling errors (Wingersky and Lord, 1984). Fink et al. (2018) used a one-sided *t*-test to examine whether the parameter estimates of a common item from the current test cycle differed significantly from the parameter estimates of the same item from the preceding test cycle.

The last step of the equating procedure, the *FCIP calibration*, involves the parameter estimation of all items using marginal maximum likelihood (MML; Bock and Aitkin, 1981) based on the responses from all test cycles. Because one aim of the CCS is to maintain the original scale from the initial calibration (first test cycle), the use of one step procedures (e.g., concurrent calibration; Wingersky and Lord, 1984) for estimating all item parameters of the different test cycles in one run is not suitable. If maintaining the scale from the initial calibration over the following test cycles has no priority, promising methods exist for equating multiple test forms simultaneously (Battauz, 2018). In the FCIP calibration, the parameters of the final common items are fixed at the item parameters estimated from the previous test cycle, whereas all the other items are estimated freely. If a “breakdown” occurs, which means that less than two common items remain after the IPD detection, a concurrent calibration (Wingersky and Lord, 1984) is used to establish a new scale.

### Specifications of the Common Item Selection

The common item selection and the scale transformation of the common items are crucial parts of the CCS because they ensure that the procedure functions well. In terms of the common item selection, different distributional assumptions such as an approximated normal distribution, as used in Fink et al. (2018), or a uniform distribution may underlie the item selection. Up to now, only Vale et al. (1981) examined the impact of different common item distributions on the accuracy of the item parameter estimates using the mean/sigma method (Marco, 1977). The authors selected the common items in such a way that the test information curves of the common items were peaked (with the most information at theta equals zero) or had an approximately normal or uniform shape. In terms of the bias of the item parameter estimates, the peaked test information curve performed worst. There were only slight differences in the performance, depending on whether normally or uniformly shaped test information curves were used for the common items. As an alternative, items with extreme difficulties (bimodal distribution) might be selected as common items for the linking cluster and, therefore, might be administered to all examinees. As a consequence, the number of responses for these items increases and the impact of the few inconsistent responses that might cause bias in the estimates and prevent later administration and parameter updating in the following test cycles would be reduced. Because the quality of the equating highly depends on the common items selected, it may be argued that especially a bimodal distribution of the common items threatens the goal of maintaining the scale across test cycles. However, the item drift test implemented in the CCS ensures that significant changes in the parameter estimates of the common items between test cycles do not affect the later FCIP calibration that is used to maintain the scale.

### Scale Transformation

When item parameters are estimated using different groups of examinees, the obtained parameters are often not comparable due to arbitrary decisions that have been made to fix the scale of the item and person parameter space (Yousfi and Böhme, 2012). In that case, the comparability of the item parameters can be attained by an IRT scale transformation. If the underlying IRT model holds for two groups of examinees, *K* and *L*, then the logistic IRT scales differ by a linear transformation for both the item parameters and the person parameters (Kolen and Brennan, 2014). The linear equation for the 𝜃-values can be formulated as follows:

(2) $$\theta_{\text{Lj}}=A\theta_{\text{Kj}}+B,$$

where *A* and *B* represent the transformation constants (also referred to as slope and shift) and 𝜃_Kj_ and 𝜃_Lj_ the person parameter values for an examinee *j* on scale *K* and scale *L*. The item parameters for the 2PL model on the two scales are defined in Eqs 3 and 4, where a_Ki_, b_Ki_, and a_Li_, b_Li_ represent the item parameters on scale *K* and on scale *L*, respectively.

(3) $$a_{\text{Li}}=\frac{a_{\text{Ki}}}{A}$$

(4) $$b_{\text{Li}}=Ab_{\text{Ki}}+B$$

To obtain the transformation constants *A* and *B*, several scale transformation methods can be used. The *moment methods* such as the mean/mean and the mean/sigma express the relationship of scales by using the means and standard deviations of item or person parameters, whereas the *characteristic curve methods* minimize a discrepancy function with respect to the item characteristic curves (Haebara, 1980) or the test characteristic curve (Stocking and Lord, 1983). Research comparing these methods has found that characteristic curve methods produced more stable results compared to the moment methods (e.g., Baker and Al-Karni, 1991; Kim and Cohen, 1992; Hanson and Béguin, 2002). Within the moment methods, the mean/mean method turned out to be more stable (Ogasawara, 2000). Furthermore, Kaskowitz and de Ayala (2001) found that characteristic curve methods were robust against moderate estimation errors and were more accurate with a larger number of common items (15 or 25 compared to only five common items). In sum, the moment methods are easily implementable, but the characteristic curve methods seem to be more robust against estimation errors.

## Research Questions

As the purpose of equating procedures in the CCS is to enable an interchangeable score interpretation across test cycles, the selection of the common items is a crucial factor for feasible equating. Up to now, only recommendations for the number of common items that should be used when conducting IRT equating have been made (Kolen and Brennan, 2014). Furthermore, it is suggested that the common items should represent the content and statistical characteristics of the test or rather the complete item pool. For example, modifying the common item selection in such a way that more items with extreme item difficulty levels are included may enhance the precision of these items, but it could threaten the quality of the equating. Therefore, our first two research questions can be formulated as follows:

1. What effect does the difficulty distribution of the common items in the CCS have on the precision of the item parameter estimates?
2. What effect does the difficulty distribution of the common items in the CCS have on the quality of the equating?

Fink et al. (2018) used the mean/mean method for scale transformation because of its simple and user-friendly implementation. Given prior research on scale transformation methods, this might not be the best choice when the sample size per test cycle is low. Furthermore, there are several packages for the open-source software R (R Core Team, 2018) available to implement the characteristic curve methods (e.g., Weeks, 2010; Battauz, 2015). As already mentioned above, the scale transformation method used and the IPD detection implemented in the CCS could serve as quality assurance to ensure that significant changes in the parameter estimates of the common items between test cycles do not affect the later FCIP calibration. For this reason, our third research question is:

3. What effect does the scale transformation method used in the CCS have on the quality of the equating?

As the CCS was developed for a context in which separate calibration studies are often not feasible and sample sizes are too low to allow for stable item parameter estimation, it is important to evaluate whether the results for these three research questions were affected by the sample size. Consequently, each of the three research questions was investigated with a special focus on additional variations of the sample size.

## Materials and Methods

### Study Design

Many factors can affect the quality of the equating within the CCS. These include, among others, the number of common items, the test length, the characteristics of the common items, the scale transformation method applied, the number of examinees per test cycle, the presence of IPD and the test applied for IPD. In the present study, some of these factors were kept constant (e.g., number of common items, test length, the presence of IPD, test applied for IPD) to ensure the comprehensibility of the study results.

To answer the research questions stated above, a Monte Carlo simulation based on a full factorial design with three independent variables (IVs) was conducted. With the first IV, *difficulty distribution*, the distribution of easiness parameters *d*_i_ of the common items (normal, uniform, and bimodal with very low and very high difficulties only) was varied. The second IV, *transformation method*, compared the most common scale transformation methods (mean/mean, mean/sigma, Haebara, and Stocking-Lord) used for computing the transformation constants to conduct the scale transformation. The third IV, *sample size*, reflected the number of test takers per test cycle (*N* = 50; *N* = 100; *N* = 300). Because the CCS uses the responses from multiple test cycles, the number of test takers per test cycle chosen for the study is small compared to the recommendations (e.g., a minimum of 500 responses per item for the 2PL model; de Ayala, 2009). The fully crossed design comprised 3 × 4 × 3 = 36 conditions. For each of the conditions, 200 replications were conducted and analyzed with regard to various evaluation criteria (see below).

The simulations were carried out in R (R Core Team, 2018) using the “mirtCAT” package (Chalmers, 2016) for simulating adaptive tests and the “mirt” package (Chalmers, 2012) for item and person parameter estimation. Transformation constants were calculated based on the common items of consecutive test cycles using the “equateIRT” package (Battauz, 2015). The test for IPD was also conducted with the “equateIRT” package. We decided to use the “equateIRT” package in the simulations because it enables a direct import of results from the “mirt” package and offers an implemented test for IPD. The corresponding functions were called in a R script, which was written to carry out the CCS.

### Simulation Procedure

#### Data Generation

In each replication, the discrimination parameters *a*_i_ were drawn from a lognormal distribution, *a*_i_ ∼ log*N* (0, 0.25), and the easiness parameters *d*_i_ were drawn from a truncated normal distribution, *d*_i_ ∼ N (0, 1.5), *d*_i_ ∈(−2.5, 2.5). Since this study was not designed to investigate IPD detection rates (e.g., Battauz, 2019), no IPD was simulated in the data. Therefore the true item parameters *a*_i_ and *d*_i_ remained unchanged over the test cycles.

The ability parameters of the examinees in the first test cycle in each replication were randomly drawn from a standard normal distribution, 𝜃 ∼ *N* (0, 1). For the subsequent test cycles *t* within a replication, the ability parameters followed a normal distribution, 𝜃 ∼ *N* (μ_t_, σ_t_), whereby the mean μ_t_ ∈ (−0.5, 0.0, 0.5) and the standard deviation σ_t_ ∈ (0.7. 1.0, 1.3) were randomly drawn. This was done to mimic the fact that examinees of different test cycles usually differ with respect to the mean and variance of their ability distribution. The examinees’ responses to the items were generated in line with the 2PL model.

#### Specification of the CCS

The CCS in the current study was applied with all seven steps proposed by Fink et al. (2018) including the IPD detection of the common items. Although no IPD was simulated in the data, in realistic settings the untested assumption of item parameter invariance is questionable. Even in the absence of IPD item parameters can significantly differ between test cycles because of sampling error. The number of test cycles within the CCS was set to 10 test cycles, whereby the first test cycle represented the initial phase and the subsequent test cycles the continuous phase. The test length was kept constant with 60 items. The calibration cluster in the continuous phase consisted of 20 items, resulting in an item pool size of I_t_ = 60 + (t − 1) ⋅ 20 after the test cycle t, and a total item pool size of 240 items after the 10th test cycle. Following the recommendation of Kolen and Brennan (2014) that the number of common items should be at least 20% of the test length, the number of common items in the linking cluster was set to 15 items. Consequently, the adaptive cluster in each test cycle of the continuous phase contained 25 items. Within the adaptive cluster, the *maximum a posteriori* (MAP; Bock and Aitkin, 1981) was used as the ability estimator and the maximum information criterion (Lord, 1980) was applied for the adaptive item selection.

For the common item selection within the equating procedure, only items that had already been calibrated in the previous test cycles and that did not serve as common items in the preceding test cycle were eligible. The selection procedure for the common items differed depending on the intended distribution. For the normal distribution, the procedure of Fink et al. (2018) was applied. The eligible items were first assigned to five categories (very low, low, medium, high, and very high) based on their easiness parameters *d*_i_. Then, five items from the “medium” category, three items each from the “low” and “high” categories, and two items from each of the extreme categories were chosen to mimic a normal distribution. For the uniform distribution, the eligible items were assigned to 15 categories based on their easiness parameters *d*_i_ and one item from each category was drawn. The interval limits of the categories were determined as quantiles of the item difficulty distribution. For the bimodal distribution, the eligible items were ordered according to their easiness parameters *d*_i_ and two subsamples were formed containing the 11 easiest and the 11 hardest items, respectively. Then, 15 items in total were randomly drawn from the two subsamples (seven easy and eight difficult items, or vice versa). As already mentioned, the selected common items in periodical assessments should be comparable also with regard to content characteristics. Content balancing approaches like the maximum priority index (Cheng and Chang, 2009) and the shadow testing approach (van der Linden and Reese, 1998) may be used for this purpose. Because no substantial impact was expected on the measurement precision of the item parameters or on the quality of the equating, content balancing was not considered as a factor in the study.

For the scale transformation, one of the four transformation methods (Mean/Mean, Mean/Sigma, Haebara, and Stocking-Lord) was applied. A modified version of Lord’s chi-squared method (Lord, 1980) that is implemented in the “equateIRT” package (Battauz, 2015) was used as the test for IPD with a type I error level of 0.05. In an iterative purification process (Candell and Drasgow, 1988) of scale transformation and testing for IPD, items that showed significant IPD were removed from the set of common items. In each test cycle, MML estimation was used to obtain the item parameters for both the temporary item parameter estimation and the FCIP calibration. The lower and the upper bound for the item discrimination *a*_i_ was set to –1 and 5, respectively. For the item easiness parameters *d*_i_, the bounds were set to –5 and 5.

### Evaluation Criteria

The mean squared error (*MSE*) of the item parameters *a*_i_ and *d*_i_, respectively, was calculated after each test cycle *t* as the averaged squared difference between the item parameter estimates and the true item parameters for all items I_t_ across all replications R = 200. Thus, a high degree of precision is denoted by low values for the *MSE*.

(5) $$MSE_{\text{t}}(a_{\text{i}})=\frac{1}{R*I_{t}}\sum_{\text{r}=1}^{R}\sum_{\text{i}=1}^{I_{\text{t}}}{\left({\overset{⌢}{a}}_{\text{ir}}-a_{\text{ir}}\right)}^{2}$$

(6) $$MSE_{\text{t}}(d_{\text{i}})=\frac{1}{R*I_{t}}\sum_{\text{r}=1}^{R}\sum_{\text{i}=1}^{I_{\text{t}}}{\left({\overset{⌢}{d}}_{\text{ir}}-d_{\text{ir}}\right)}^{2}$$

Because our aim was to evaluate whether the modified common item selection could prevent a dysfunction of the CCS in terms of more precise item parameter estimates for items with very low and very high values for *d*_i_, the conditional *MSE* was used as a criterion. Therefore, the *MSE* was calculated for seven easiness intervals: *d*_i_ ∈ (−*Inf*,−2], *d*_i_ ∈ (−2,−1], *d*_i_ ∈ (−1, −0.25], *d*_i_ ∈ (−0.25, −0.25], *d*_i_ ∈ (−0.25, −1], *d*_i_ ∈ (1, 2], and *d*_i_ ∈ (2, *Inf*).

Three criteria were used to evaluate the equating quality. As a first criterion, we used the proportion of test cycles in which no breakdown of the common items occurred. Second, we calculated the proportion of drifted items for each of the 36 conditions. And third, we computed the accuracy (*Error*) of the scale transformation constants *A* and *B* for each replication r when no breakdown occurred as the difference between the true and the estimated transformation constants for every test cycle in the continuous phase. The average of the *Error* corresponds to the Bias of the transformations constants.

(7) $$Error\text{ (}A_{\text{tr}})=\left({\overset{⌢}{A}}_{\text{tr}}-A_{tr}\right)$$

(8) $$Error\text{ (}B_{\text{tr}})=\left({\overset{⌢}{B}}_{\text{tr}}-B_{tr}\right)$$

The true transformation constants *A* and *B* were calculated based on the true examinees’ abilities from/in all previous test cycles p and from/in the current test cycle *t* (Kolen and Brennan, 2014).

(9) $$A_{\text{t}}=\frac{\sigma \left(\theta_{\text{t}}\right)}{\sigma \left(\theta_{\text{p}}\right)}$$

(10) $$B_{\text{t}}=\mu (\theta_{\text{t}})-A\mu (\theta_{\text{p}})$$

The estimated transformation constants ${\hat{A}}_{\text{t}}$ and ${\hat{B}}_{\text{t}}$ were obtained based on the parameter estimates of the final set of common items from the previous and the current test cycles using one of the four scale transformation methods implemented in the “equateIRT” package (Battauz, 2015). The third criterion was calculated only for the cases where at least two common items remained after the IPD detection.

## Results

Note that the conditions with the mean/mean method as scale transformation method and normal distributed common items mimic the setup of the equating procedure from Fink et al. (2018).

### Conditional Precision of Item Parameters

**FIGURE 1:**
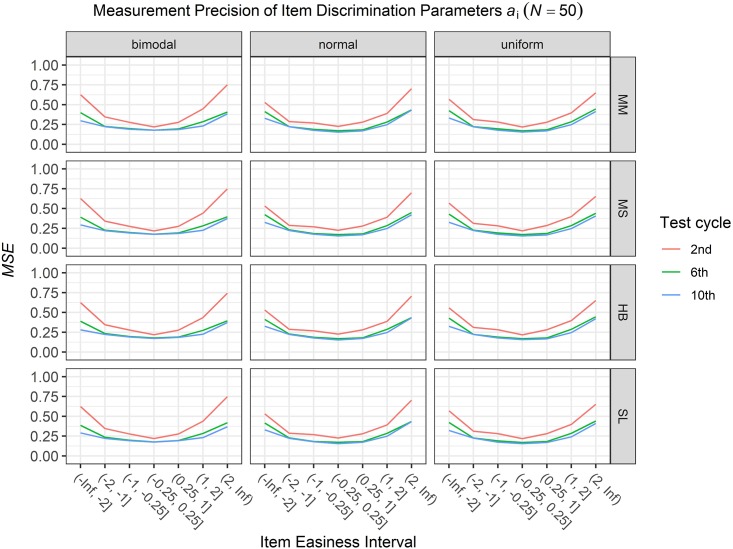
Conditional mean squared error (*MSE*) of the item discrimination *a*_i_ for specific item easiness intervals after the 2nd, 6th, and 10th test cycles in the continuous calibration strategy with a sample size per test cycle of *N* = 50 for different common item difficulty distributions and different scale transformation methods (MM = Mean/Mean, MS = Mean/Sigma, HB = Haebara, SL = Stocking-Lord).

**FIGURE 2:**
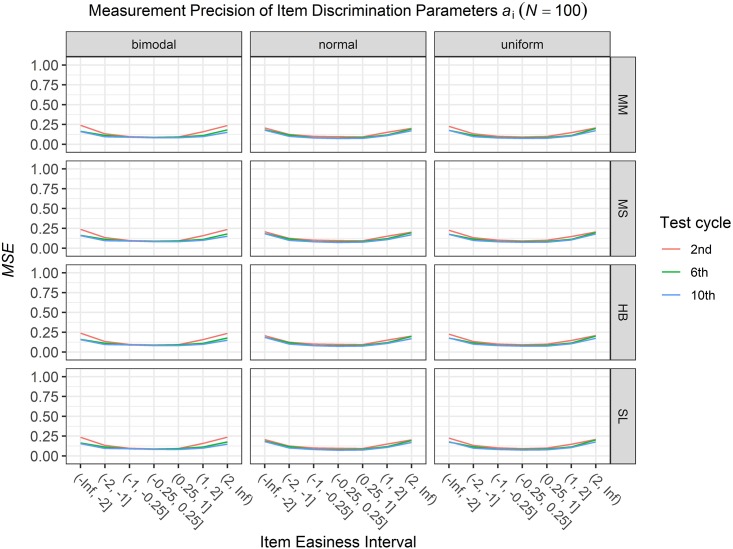
Conditional mean squared error (*MSE*) of the item discrimination *a*_i_ for specific item easiness intervals after the 2nd, 6th, and 10th test cycle in the continuous calibration strategy with a sample size per test cycle of *N* = 100 for different common item difficulty distributions and different scale transformation methods (MM = Mean/Mean, MS = Mean/Sigma, HB = Haebara, SL = Stocking-Lord).

**FIGURE 3:**
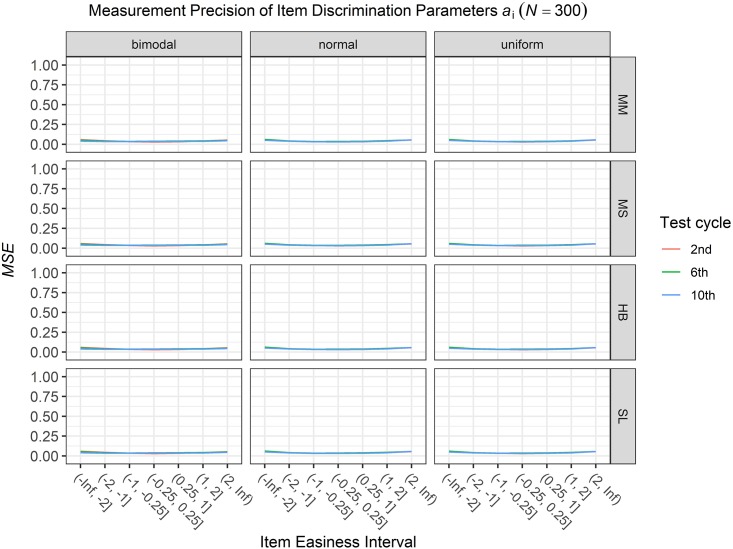
Conditional mean squared error (*MSE*) of the item discrimination *a*_i_ for specific item easiness intervals after the 2nd, 6th, and 10th test cycle in the continuous calibration strategy with a sample size per test cycle of *N* = 300 for different common item difficulty distributions and different scale transformation methods (MM = Mean/Mean, MS = Mean/Sigma, HB = Haebara, SL = Stocking-Lord).

**FIGURE 4:**
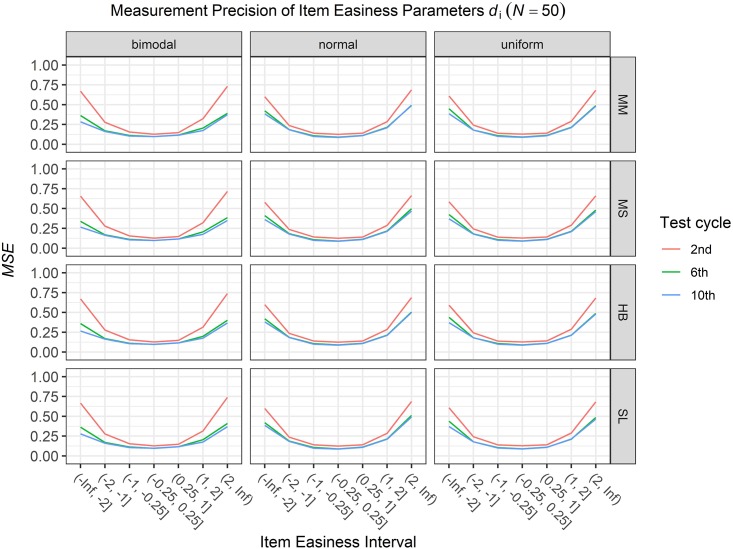
Conditional mean squared error (*MSE*) of the item easiness *d*_i_ for specific item easiness intervals after the 2nd, 6th, and 10th test cycle in the continuous calibration strategy with a sample size per test cycle of *N* = 50 for different common item difficulty distributions and different scale transformation methods (MM = Mean/Mean, MS = Mean/Sigma, HB = Haebara, SL = Stocking-Lord).

**FIGURE 5:**
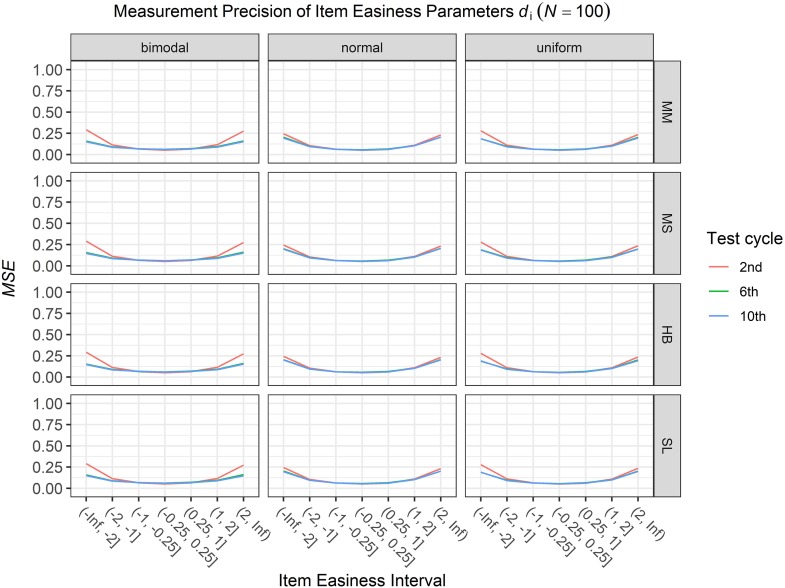
Conditional mean squared error (*MSE*) of the item easiness *d*_i_ for specific item easiness intervals after 2nd, 6th, and 10th test cycle in the continuous calibration strategy with a sample size per test cycle of *N* = 100 for different common item difficulty distributions and different scale transformation methods (MM = Mean/Mean, MS = Mean/Sigma, HB = Haebara, SL = Stocking-Lord).

**FIGURE 6:**
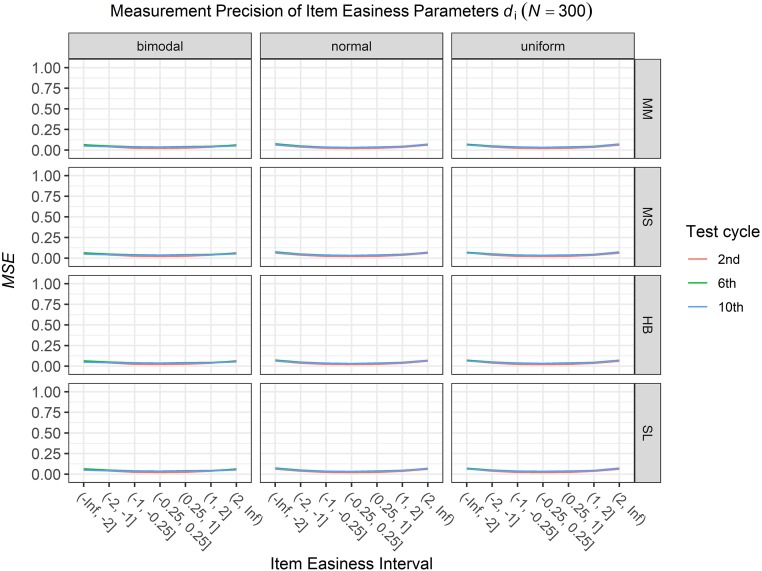
Conditional mean squared error (*MSE*) of the item easiness *d*_i_ for specific item easiness intervals after the 2nd, 6th, and 10th test cycle in the continuous calibration strategy with a sample size per test cycle of *N* = 300 for different common item difficulty distributions and different scale transformation methods (MM = Mean/Mean, MS = Mean/Sigma, HB = Haebara, SL = Stocking-Lord).

To answer the first research question regarding the precision of the item parameter estimates, we analyzed the conditional *MSE* of the item discrimination parameters *a*_i_ and the item easiness parameters *d*_i_ depending on the scale transformation method, the common item difficulty distribution, and the sample sizes per test cycle. For the sake of clarity, the results are only presented for the second, the sixth, and the 10th test cycles of the CCS. Figure 1–3 illustrate the conditional *MSE* of the item discrimination parameter estimates *a*_i_, and Figure 4–6 illustrate the conditional *MSE* of the item easiness parameter *d*_i_. As can be expected based on the findings from Fink et al. (2018), the *MSE* for the item discrimination parameter estimates and the item easiness parameter estimates decreased as the number of test cycles in the CCS increased and as the sample size per test cycle increased. With regard to the precision of the item parameter estimates, no substantial differences were found between the different scale transformation methods, independent of the common item difficulty distribution and the sample size per test cycle. When a bimodal difficulty distribution of common items was chosen, the precision of the item parameter estimates for the very easy and very difficult items was higher compared to a normal or uniform difficulty distribution of common items (Figure 1, 4). However, this minimal gain came at the expense of a lower precision of the item parameter estimates for items with medium difficulty. This effect was found for very small sample sizes per test cycle (*N* = 50), and diminished for larger sample sizes (*N* = 100, *N* = 300).

### Quality of Equating

The second and third research questions focused on the equating procedure. The first evaluation criterion was the proportion of feasible equatings (at least two items remained after the IPD detection). Most striking was that over all replications for none of the test cycles a breakdown of the common items occurred. Furthermore, for all 36 conditions the median number of eligible common items over all test cycles and replications ranged from 14 to 15.

**FIGURE 7:**
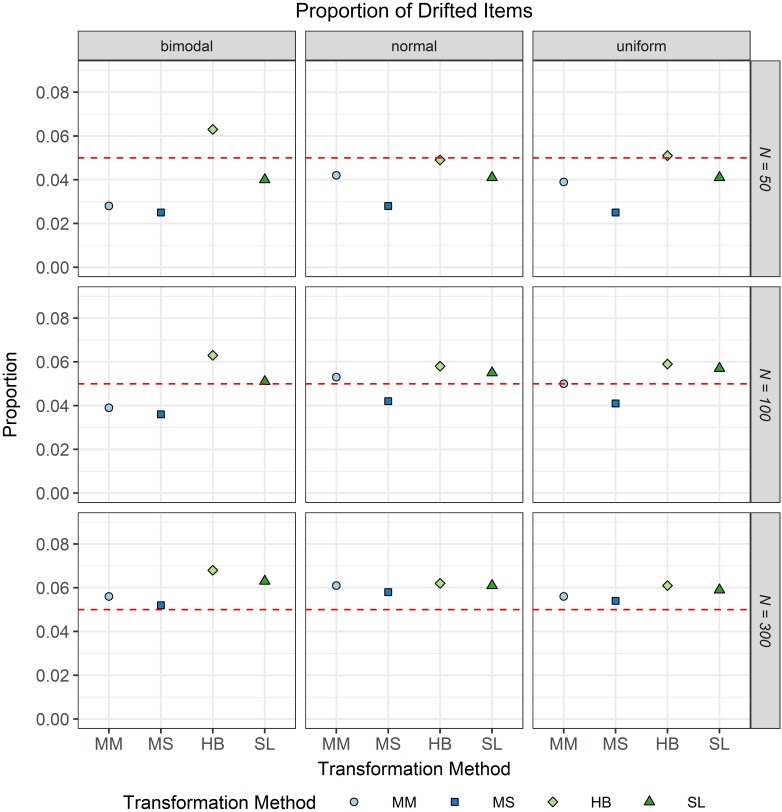
Proportion of drifted items in the continuous calibration strategy for different sample sizes per test cycle, different common item difficulty distributions, and different scale transformation methods (MM = Mean/Mean, MS = Mean/Sigma, HB = Haebara, SL = Stocking-Lord). The dashed line represents the type I error level of 0.05.

The second evaluation criterion was the proportion of drifted items. As IPD was not simulated in the study and because the type I error level of the test for IPD was set to 0.05, it was expected that approximately five percent of the common items would show significant IPD. Figure 7 shows the proportion of drifted common items depending on the common item difficulty distribution, the scale transformation method, and the sample size per test cycle. It is obvious from this figure that independent of the scale transformation method and the common item difficulty distribution, the type I error rates increased with increasing sample size per test cycle. This effect was stronger for the moment/methods. Furthermore, it became apparent that if the difficulty distribution of the common items was uniform or normal, all scale transformation methods did not considerably differ from the type I error level of 0.05. The only exception to this result was the mean/sigma method which generally led to considerably smaller type I error rates when the sample size was small (*N* = 50). All in all, using the Stocking-Lord method resulted for all conditions in type I error rates that did not considerably differ from the type I error level of 0.05.

**FIGURE 8:**
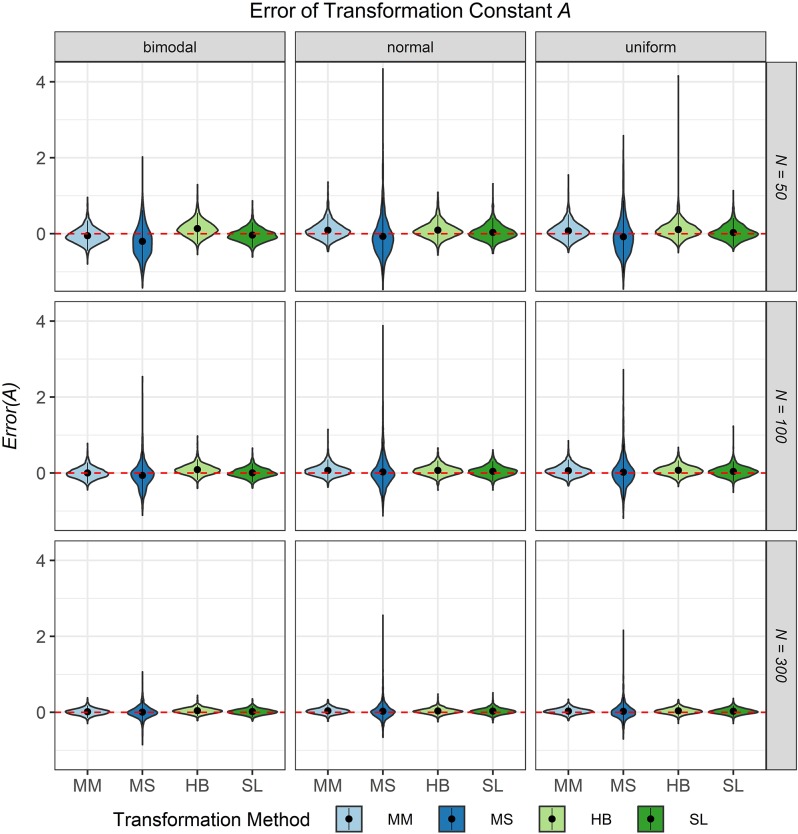
Error of the transformation constant *A* in the continuous calibration strategy for different sample sizes per test cycle, different common item difficulty distributions, and different scale transformation methods (MM = Mean/Mean, MS = Mean/Sigma, HB = Haebara, SL = Stocking-Lord). The dot in the middle of each violin represents the bias and the width of the violin expresses the frequency of the corresponding value.

The third evaluation criterion was the accuracy of the transformation constants *A* and *B* when no breakdown occurred. Figure 8, 9 show violin plots for the *Error* of the transformation constants *A* and *B* depending on the common item difficulty distribution, the scale transformation method, and the sample size per test cycle. In violin plots, the frequency distribution of a numeric variable (e.g., bias) is expressed. Note that the average error ( = *Bias*; represented by the dot in the violin) for both transformation constants *A* and *B* did not differ substantially from zero for all scale transformation methods, independent of the common item difficulty distribution and the sample size per test cycle. However, the variation of the error (represented by the height of the violin) differed between the scale transformation methods and, especially for the moment methods rather high levels of error occurred. The characteristic curve methods showed the lowest variation in error. With increasing sample size per test cycle, the variation of the error decreased, but there were still extreme levels of error for the mean/mean and the mean/sigma method.

**FIGURE 9:**
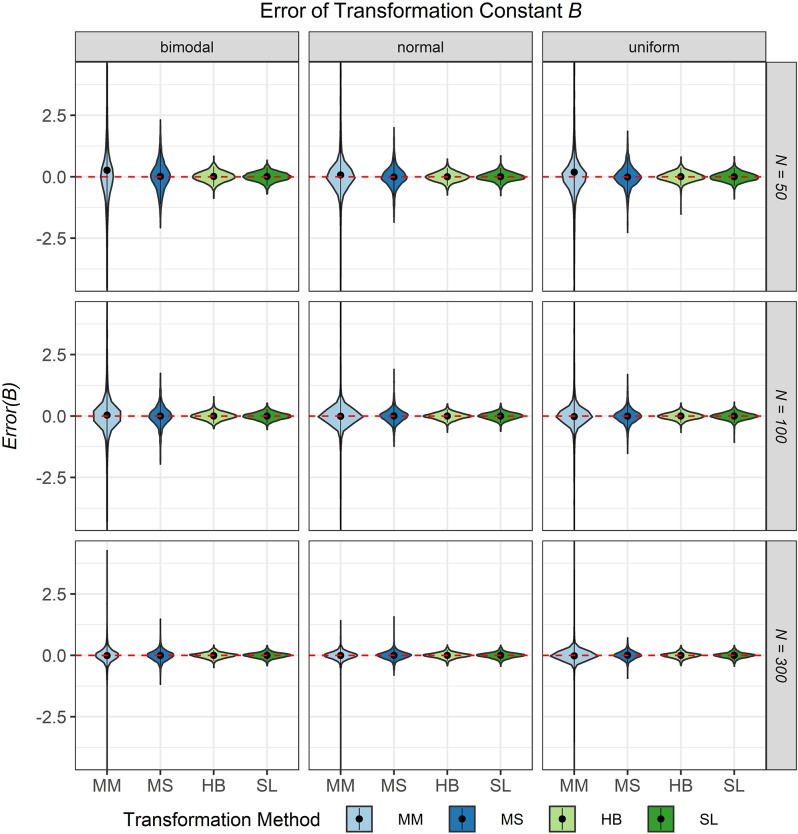
Error of the transformation constant *B* in the continuous calibration strategy for different sample sizes per test cycle, different common item difficulty distributions, and different scale transformation methods (MM = Mean/Mean, MS = Mean/Sigma, HB = Haebara, SL = Stocking-Lord). The dot in the middle of each violin represents the bias and the width of the violin expresses the frequency of the corresponding value.

In summary and in terms of the three research questions, the study provided the following results:

1. The difficulty distribution of the common items in the CCS did not have a substantial impact on the precision of the item parameter estimates although small differences existed between the common item distributions; these differences were in opposite/varying directions for extreme and medium-ranged item easiness parameters *d*_i_ when the sample size was very small.
2. With regard to the proportion of feasible equatings (at least two common items remained after the test for IPD) no differences were found independent of the common item difficulty distributions, the scale transformation method and the sample size.
3. The characteristic curve methods outperformed the moment methods in terms of error of the transformation constant. Especially for small sample size the mean/sigma method cannot recommended.

## Discussion

The objective of the present study was to evaluate different setups of the equating procedure implemented in the CCS and to make/provide recommendations on how to apply these setups. For this purpose, the quality of the item parameter estimates and of the equating was examined in a Monte Carlo simulation for different common item difficulty distributions, different scale transformation methods, and different sample sizes per test cycle.

The following recommendations can be made based on the results obtained: First, no clear advantage of using any of the three common item difficulty distributions was identified. Regarding the precision of the item parameter estimates, the results show a slight increase in the precision of the item parameter estimates for items with extreme difficulties when using a bimodal common item difficulty distribution compared to a normal or uniform distribution. However, the precision of the item parameter estimates for items with medium difficulty decreased. These effects were only found for very small sample sizes per test cycle (*N* = 50) and no differences were found for larger sample sizes (*N* = 100, *N* = 300). Furthermore, the use of different scale transformation methods did not have a substantial effect on the precision of the item parameter estimates.

Note that exposure control methods (e.g., Sympson and Hetter, 1985; Revuelta and Ponsoda, 1998; Stocking and Lewis, 1998) might be an alternative to increase the number of responses to items with extreme difficulty levels and, in consequence, the precision of the item parameter estimates for these items. However, using these methods would sacrifice adaptivity to a certain degree and, thus, the efficiency of the computerized adaptive test (e.g., Revuelta and Ponsoda, 1998). This is even more relevant to tests assembled within the partly adaptive CCS, because only one of the three cluster types used is based on an adaptive item selection. Furthermore, in the early stages of the CCS, the item pool is rather small, which also limits the adaptivity of the tests. For these reasons, it can be expected that exposure control methods do not offer an ideal option for the CCS to increase the precision of item parameter estimates for items with extreme difficulties. This point might be examined by future research.

Second, with respect to the quality of the equating, no difference was found for the scale transformation methods with regard to the proportion of feasible equatings independent of the common item difficulty distribution used and the sample size available per test cycle. The rule for evaluating an equating as feasible (at least two common items remained after the test for IPD) is worthy of discussion because of two reasons: first, with a small number of remaining common items, the equating procedure is more prone to sampling error (Wingersky and Lord, 1984) and second, it is rather unlikely that the content of the item pool is adequately reflected by the remaining common items. However, even if the criterion for evaluating an equating as feasible had been set to ten remaining common items, the proportion of feasible equatings would be at least 99% in all conditions. With regard to the type I error rate and the error of the transformation constant the characteristic curve methods outperformed the moment methods especially for small sample sizes. This is in line with the result of Ogasawara (2002) who found that the characteristic curve methods are less affected by imprecise item parameter estimates and lead to more accurate transformation than moment methods. Among the characteristic curve methods the Stocking-Lord method was slightly better than the Haebara method in almost all conditions. Thus, although our results do not facilitate a clear recommendation regarding the most favorable common item difficulty distribution, they do enable a clear recommendation in terms of the preferred scale transformation method: The Stocking-Lord method should be used as the scale transformation method within the CCS.

## Author Contributions

SB conceived the study, conducted the statistical analyses, drafted the manuscript, and approved the submitted version. AFi performed substantial contribution to the conception of the study, contributed to the programming needed for the simulation study (R), reviewed the manuscript critically for important intellectual content, and approved the submitted version. CS performed substantial contributions to the interpretation of the study results, reviewed the manuscript critically for important intellectual content, and approved the submitted version. AFr provided advise in the planning phase of the study, reviewed the manuscript critically for important intellectual content, and approved the submitted version.

## Conflict of Interest Statement

The authors declare that the research was conducted in the absence of any commercial or financial relationships that could be construed as a potential conflict of interest. The handling Editor declared a shared affiliation, though no other collaboration, with one of the authors AFr at the time of review.
